# Sociability of male cancer patients with stoma: a qualitative metasynthesis

**DOI:** 10.1590/0034-7167-2025-0021

**Published:** 2025-12-08

**Authors:** João Vitor Antunes Lins dos Santos, Ariana Luiza Rabelo, Vander Monteiro da Conceição, Luís Fernando Santos Corrêa da Silva, Anderson Reis de Sousa, Jeferson Santos Araújo

**Affiliations:** I Universidade Federal da Fronteira Sul. Erechim, Rio Grande do Sul, Brazil; II Universidade Federal da Bahia. Salvador, Bahia, Brazil; III Universidade Federal da Fronteira Sul. Chapecó, Santa Catarina, Brazil

**Keywords:** Men’s Health, Ostomy, Social Behavior, Neoplasms, Systematic Review., Salud del Hombre, Estomía, Conducta Social, Neoplasias, Revisión Sistemática.

## Abstract

**Objectives:**

to synthesize evidence from qualitative studies produced in literature on the sociability of men with ostomies due to a cancer diagnosis.

**Methods:**

a qualitative metasynthesis was conducted in seven databases with studies published between 2004 and 2024. The sample included six studies, analyzed using MaxQDA^®^ software, validated by Critical Appraisal Skills Programme, and synthesized based on the concept of sociability.

**Results:**

four descriptive topics were identified: a) Challenges in sexual performance; b) Physical and emotional impacts: self-esteem and social isolation; c) Social limitations and strategies for living with a stoma in society; d) Alternating male behaviors within the family structure. These topics were encompassed by the analytical topic: Male sociability in transformation: experiences of men with a stoma.

**Final Considerations:**

culturally constructed masculinity shapes men’s sociability, but living with a stoma generates insecurity, redefines social roles, and significantly impacts the dynamics of public and private relationships.

## RESUMEN

**Objetivos:**

sintetizar la evidencia de estudios cualitativos publicados en la literatura sobre la sociabilidad de hombres ostomizados por cáncer.

**Métodos:**

se realizó una metasíntesis cualitativa en siete bases de datos con estudios publicados entre 2004 y 2024. La muestra incluyó seis estudios, analizados con el software MaxQDA^®^, validados por el Critical Appraisal Skills Programme y sintetizados con base en el concepto de sociabilidad.

**Resultados:**

se identificaron cuatro temas descriptivos: a) Desafíos en el desempeño sexual; b) Impactos físicos y emocionales: autoestima y aislamiento social; c) Limitaciones sociales y estrategias para vivir con una ostomía en sociedad; d) Comportamientos masculinos alternantes dentro de la estructura familiar. Estos temas se englobaron en el tema analítico: Sociabilidad masculina en transformación: experiencias de hombres con ostomía.

**Consideraciones Finales:**

la masculinidad culturalmente construida moldea la sociabilidad de los hombres, pero vivir con una ostomía genera inseguridad, redefine los roles sociales e impacta significativamente la dinámica de las relaciones públicas y privadas.

## INTRODUCTION

Despite advances in its treatment, cancer remains a significant public health problem worldwide. In 2020, there were a total of 19,292,789 new cases of the disease globally, resulting in 9,958,133 deaths^(1)^. This diagnosis entails physical and social repercussions due to the adverse effects of treatment, which significantly transform their bodies and lives^(2)^.

A study^(3)^ emphasizes that, globally, historical and cultural processes associated with masculine identity are linked to ideals of virility, power, and hierarchy in gender relations. These factors shape the behavior of this population, distancing them from seeking preventive care. Furthermore, there are social demands that make up the essence of masculinity and require the pursuit of autonomy, strength, and control of emotions and feelings as criteria for individual’s full masculinity. However, these ideals hinder the early diagnosis of silent diseases, especially bowel and urinary tract cancer, which often require the creation of an elimination stoma^(4)^. Therefore, this issue, of great relevance to public health, highlights the importance of essential actions for professional practice in the field of nursing.

The scientific literature^(5,6)^ has also highlighted that cultural and identity representations associated with cancer and masculinity are influenced by the social idealization of the male figure, which can significantly affect how men cope with and deal with the disease, impacting how they care for their bodies, maintain their beliefs, and relate to health. Furthermore, they establish links between masculinity and the cultural context, in the pursuit of an idealized pattern of hierarchical dominance over other men and women, with behaviors that deny the embodiment of illness, weakening, and vulnerability^(3)^. Therefore, it is essential that nursing teams be able to address the relational aspects of gender in the health and disease processes, and the clinical management of health demands and needs.

The creation of an elimination stoma generates physical, psychological, and social impacts. Physical limitations in men, especially cisgender men, include sexual disorders, including erectile dysfunction, changes in sex drive, and changes in arousal, libido, and fertility. Psychological repercussions may include shame, discomfort, fear, loneliness, and other factors, which can have social repercussions, such as separation from family and work, and social isolation^(7)^.

For men, these challenges become a source of fragility in their role in the family and society^(3)^, which affect social reintegration and include restrictions imposed by the procedure, such as the use of public restrooms, problems related to odor and overflow, as well as limitations in the practice of physical exercise and leisure^(8)^. Faced with this vulnerability, men undergo transformations in their bodies and identities throughout their lives, adopting alienating behaviors in relation to their own bodies due to feelings of difference, making it a challenge to interact with other people^(4,9)^.

Therefore, the sociability of men with ostomies plays a crucial role as an analytical concept, as it is necessary to understand the phenomenon as a complex set of social practices and interactions that permeate the social and health fields. Therefore, it requires specialized attention from nursing and healthcare professionals, which justifies this study. Sociability is understood as the structure of individual and collective dispositions of social agents, the symbolic systems, and the social structures in which they are inserted. Thus, it goes beyond superficial interactions between individuals, encompassing power relations, symbolic struggles, and social and identity hierarchies^(10)^.

Researchers^(11)^ also highlight the alarming decline in sociability among middle-aged people in Asian countries and others such as the United States, France, Italy, Spain, and Australia. The effects of this seclusion have a direct impact on human health: men with low levels of sociability have a higher risk of mortality from chronic degenerative diseases, such as cancer. Given this fact, it becomes clear that social relationships, support networks, and social context are fundamental to physical and mental well-being, influencing perspectives, behaviors, and even opportunities for accessing healthcare and support^(4,9)^.

Although not yet synthesized, qualitative evidence exists that describes the phenomenon and can help nurses and healthcare professionals provide comprehensive care to men with stoma. This reveals a significant gap in scientific knowledge, which can be addressed with the findings summarized here. Thus, given the context presented, in which living with a stoma encompasses various aspects of human subjectivity, involving individual and collective perceptions and meanings, for the field of nursing, research like this can inspire new approaches to the care of male patients facing cancer and stoma, marking a transformative phase in their lives due to the uniqueness of their conditions.

## OBJECTIVES

To synthesize evidence from qualitative studies produced in the literature on the sociability of men with ostomies due to a cancer diagnosis.

## METHODS

### Study design

This qualitative systematic review followed the four-stage meta-synthesis methodology^(12)^: (1) comprehensive and systematic literature search, with retrieval of relevant studies; (2) quality assessment of included studies; (3) classification of findings; and (4) preparation of a meta-synthesis of results. Qualitative meta-synthesis is a rigorous and methodical process that aims to identify, abstract, and synthesize qualitative data to answer a specific research question.

The Enhancing Transparency in Reporting the Synthesis of Qualitative Research was used to report the essential elements that should make up a synthesis of qualitative evidence^(13)^. The protocol for this metasynthesis was registered with the International Prospective Register of Systematic Reviews, under Protocol CRD42024624100.

### Search strategy

To develop the research question and the search strategy, the SPIDER model^(14)^ was used. To identify eligible studies in the literature, a systematic search was conducted by two independent researchers (first and last author) in seven databases in the following order of access: US National Library of Medicine (PubMed); Cumulative Index to Nursing and Allied Health Literature (CINAHL); Excerpta Médica dataBASE (Embase); Elsevier Science’s MegaSource (ScienceDirect); Science Citation Indexes (Web of Science); American Psychological Association (APA) PsycINFO; and Latin American and Caribbean Literature in Health Sciences (LILACS). The search strategy was developed by combining terms from the Health Sciences Descriptors, Medical Subject Headings (MeSH), Elsevier’s authoritative life science thesaurus, and APA Thesaurus of Psychological Index Terms, as well as thematic keywords. The terms were associated using the Boolean operators OR and AND, according to the specificities of each database. For instance, Chart 1 presents the advanced search strategy in the PubMed database following the SPIDER model, from which the other strategies were adapted according to the specificities of each database.

**Chart 1:** SPIDER model of operationalization of metasynthesis

Acronym	Example	Search strategy
S	Men with stoma diagnosed with cancer	“Men”[MeSH] OR “male” OR “masculinity” AND “Surgical Stomas”[MeSH] OR “Ostomy”[MeSH] OR “ostomy” OR “stoma” OR “colostomy” OR “ileostomy” OR “nephrostomy” AND “Neoplasms”[MeSH] OR “cancer” OR “oncology” OR “neoplasm”
PI	Sociability	“Social Support”[MeSH] OR “social interaction” OR “sociability” OR “social experience” OR “interpersonal relations”
D	Qualitative methods	“Focus Groups”[MeSH] OR “Focus Group” OR “Group, Focus” OR “Anthropology, Medical”[MeSH] OR “Medical Anthropology” OR “Grounded Theory”[MeSH] OR “Theory, Grounded” OR “Hermeneutics”[MeSH] OR Hermeneutic OR Ethnographic OR “ethnographic research” OR Phenomenology OR “phenomenological research” OR Narrative OR “Interviews as Topic”[MeSH] OR Interviewers OR Interviewer OR Interviewees OR “Group Interviews” OR “Group Interview” OR “Interview, Group” OR “Interviews, Group” OR “in-depth interview” OR “qualitative interview” OR “content analysis” OR “semantic analysis”
E	Perspective or experience on the phenomenon	“Experience” OR “experiences” OR “sense” OR “meaning” OR “perspectives” OR “subjectivity” OR “life experiences”
R	Qualitative evidence	“Qualitative Research”[MeSH] OR “Research, Qualitative” OR “Qualitative studies” OR “Qualitative” OR “Empirical Research”[MeSH] OR “Research, Empirical”

Thus, the following research question was formulated: what evidence is there from qualitative studies on the sociability experience of men with stoma diagnosed with cancer?

**Figure 1:**
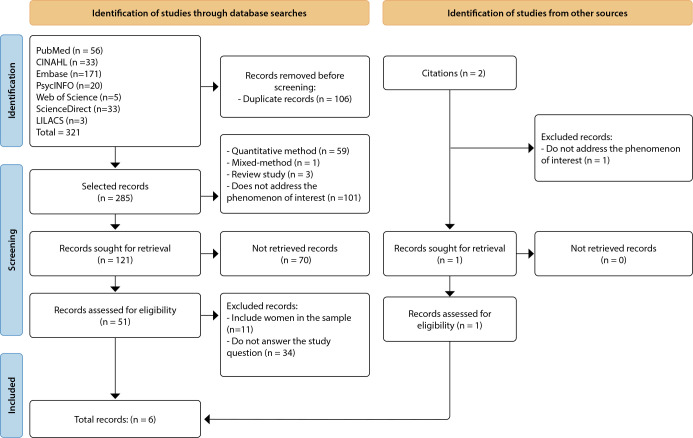
Metasynthesis operationalization flowchart according to the Preferred Reporting Items for Systematic Reviews and Meta-Analyses

The search was conducted independently by the first and last authors in September 2024, limited to recovering records available for the 20-year period (2004-2024) and conducted according to the stages proposed by the Preferred Reporting Items for Systematic Reviews and Meta-Analyses (PRISMA) flowchart^(15)^, shown in Figure 1.

### Study selection and inclusion and exclusion criteria

Through the systematic search, 321 records were identified: 56 in the PubMed database, 33 in CINAHL, 171 in Embase, 20 in PsycINFO, five in Web of Science, 33 in ScienceDirect, and three in LILACS. Two references were obtained through an unsystematic search, resulting in 321 publications.

To optimize the process, two researchers (first and last authors) independently selected the studies using the Rayyan^®^ platform^(16)^. The process was blinded, and any disagreements were resolved through consultation with a third reviewer (third author). Initially, the identified records were uploaded to the platform, and after removing duplicates, 285 were analyzed by reading the titles and abstracts. The selection criteria were then applied, and 164 records were excluded for reasons such as studies using quantitative or mixed-methods (qualitative-quantitative), review studies, and for not addressing the phenomenon of interest. In this process, 51 records were selected and analyzed in full. Among these, 11 studies were excluded because they included both male and female samples, and 34 evidence items were excluded because they did not address the research question. Finally, two references were found through other sources (citations from the selected studies), and only one was included in the study. This study included original references written in Portuguese, English, or Spanish that addressed sociability among men with ostomies due to urinary and intestinal cancer. Publications that used the qualitative method and presented the individualized discourses of the male population, published from January 2004 to October 2024, were selected.

Literature considered gray, such as books, undergraduate papers, theses and dissertations, as well as editorials, letters to the editor, reflective studies, and review papers, was excluded. It is worth noting that, in keeping with the database search order, duplicate records were considered only once. After analyzing their compliance with the inclusion and exclusion criteria, six studies were selected for the final synthesis.

### Methodological quality assessment

Following the Critical Appraisal Skills Programme criteria^(17)^, two researchers independently assessed the quality of selected studies. After analysis, the references were classified into two categories: A and B. Category A encompasses references with a low risk of bias that met nine or more of the ten criteria assessed. Category B comprises references with a moderate risk of bias that met at least five of the ten criteria analyzed. In this analytical process, the selected articles met most of the criteria established by the instrument, representing a low risk of bias. Chart 2 illustrates the assessment of selected studies by year, organized by the first author. It is important to emphasize that, during this process, no references were excluded.

**Chart 2:** Assessment of the quality of included studies according to the Critical Appraisal Skills Programme

Question	Araújo, 2022^(9)^	Neris, 2020^(18)^	Bigun, 2015^(19)^	Shipp, 2015^(20)^	Dázio, 2009^(21)^	Kandemi,2017^(22)^
1. Were the research objectives clearly stated?	•	•	•	⊕	•	•
2. Is the qualitative methodology appropriate?	•	•	•	•	•	•
3. Was the research design adequate to achieve the proposed objectives?	•	•	•	•	•	•
4. Was the recruitment strategy appropriate to the research objectives?	•	•	•	•	•	•
5. Was the data collected to address the research question?	•	•	•	•	•	•
6. Was the relationship between the researcher and the participants adequately considered?	⊕	•	•	•	•	•
7. Were ethical issues considered?	•	•	•	•	•	•
8. Was the data analysis sufficiently rigorous?	•	•	•	⊕	•	•
9. Were the results reported clearly?	•	•	⊕	•	⊕	•
10. Does the research make contributions?	•	•	•	•	•	•
- Classification -	A	A	A	A	A	A

*• - Yes; o - No;* ⊕ *- Paritally.*

### Data extraction and synthesis preparation

To collect evidence data, an instrument adapted from the JBI was used, including variables such as title, authors, country, year, place of publication, phenomenon of interest, study objectives, population and methods^(23)^. Based on this information, evidence characteristics were analyzed from a comprehensive perspective using MaxQDA^®^ software^(24)^. This software enabled expanded knowledge by exploring the extracted content in greater depth and constructing initial analysis categories. The aforementioned characteristics allowed for the synthesis of the main research findings, taking into account their particularities and identifying first-order concepts. These concepts were then compared and grouped using an interpretative approach, resulting in second-order concepts^(25)^.

Subsequently, the results were analyzed based on inductive thematic analysis^(26)^, following the following proposed stages: familiarization with the data; code generation; search for topics; continuous topic review; topic definition; and explanatory interpretation production. To construct the interpretative synthesis, presented in this study through descriptive topics to better understand the perspectives and experiences of men with stomas in their sociability process, second-order concepts were analyzed and a third-order interpretative synthesis was developed on the phenomenon studied.

## RESULTS

This meta-synthesis encompasses the description of the results of six qualitative studies. The final sample was organized and classified by title, country and year of publication, main objectives, population, and research context, as illustrated in Chart 3.

**Chart 3:** Characterization of selected studies

Title	Country/year	Objective	Sample	Method	Main results	Critical Appraisal Skills Programme quality assessment
Sexuality of men experiencing intestinal ostomies: stories about feelings and meanings^(9)^	Brazil 2022	Understand the feelings and meanings of sexuality of adult men with intestinal stomas.	30 men	Qualitative study of the historical-analytical and comprehensive-exploratory type, analyzed using the thematic oral history method.	Adult men with intestinal stomas experience multiple feelings linked to their sexuality, such as impulsive, emotional, affective and orientational feelings.	A
“What I was and what I am”: A qualitative study of survivors’ experience of urological cancer^(18)^	Brazil 2020	Analyze the experience of surviving urological cancer from men’s perspective.	Ten men	Qualitative study, using narrative methodology, analyzed based on cultural concepts derived from interpretive medical anthropology.	The narratives reveal identity transitions from healthy men to men marked by illness. In an attempt to preserve their masculinity, they sought to portray themselves as normal men, although survival represented an experience of liminality and biographical rupture.	A
A qualitative study exploring male cancer patients’ experiences with percutaneous nephrostomy^(19)^	Denmark 2015	Describe how nephrostomy is perceived by patients and its repercussions on their daily lives.	Ten men	Qualitative study, with semi-structured interviews interpreted based on Grounded Theory.	Nephrostomy treatment reduced physical activity and restricted social life. Patients reported limitations in carrying out their usual social activities, citing leakage and odor as limiting factors.	A
The impact of colorectal cancer on leisure participation: A narrative study^(20)^	Australia 2015	Explore changes in participation in leisure activities as a result of a cancer diagnosis and subsequent treatments, as well as the factors that facilitated or restricted this participation and the meaning participants attributed to leisure participation patterns.	Four men	Qualitative study, with narrative method analyzed through thematic analysis.	During treatment, men prioritized passive occupations over more active or community-based ones, due to the limitations imposed by the colostomy bag.	A
The meaning of being a man with intestinal stoma due to colorectal Cancer: an anthropological approach to masculinities^(21)^	Brazil 2009	Analyze the meanings that men with intestinal stoma attribute to the experience of the disease and treatment of colorectal cancer.	16 men	Qualitative study, with data collected through participant observation and semi-structured interviews, analyzed using an inductive approach.	The diagnostic tests left physical and emotional scars, and scratches on their masculinities. The attempt to return to normality was based on the symbolic concept of honor that governs men’s expectations and actions at home and in social life.	A
‘Sexual Problems of Patients with Urostomy: A Qualitative Study^(22)^	Turkey 2017	Identify the experiences, perceptions and problems of patients who underwent urostomy due to bladder cancer in relation to the effects of urostomy on their sexual life and that of their spouses/partners.	Ten men	Qualitative study with a phenomenological approach. Data were collected through semi-structured interviews and coded and organized into topics based on content analysis.	Men reported discomfort with their altered body image due to the stoma, noting bulge under their clothes, difficulty wearing pants, and constant use of tracksuits. They also reported sexual dysfunction and a lack of professional support to address these issues.	A

**Figure 2:**
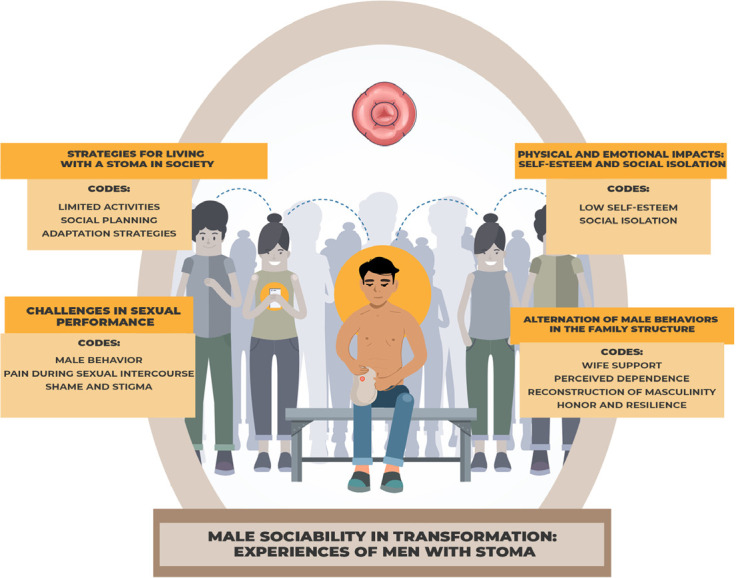
Representation of the process of topic analysis and production

The organization of first-order concepts is described based on a synthesis of the main concepts and ideas found in the original articles. Thematic synthesis allowed us to identify four descriptive topics: (a) Challenges in sexual performance; (b) Physical and emotional impacts: self-esteem and social isolation; (c) Social limitations and strategies for living with a stoma in society; (d) Alternation of male behaviors within the family structure. These descriptive topics were understood through an analytical topic entitled “Male sociability in transformation: experiences of men with stoma”. Figure 2 illustrates the process of topic analysis and production.

The analytical topic reveals that changes in male sociability are experienced with emotional distress related to treatment, rehabilitation, and adaptation to a new lifestyle. Men describe the experience as a loss of autonomy and virility, feeling limited in their social and emotional identity, as if they were losing the attributes traditionally associated with masculinity.

### Physical and emotional impacts: self-esteem and social isolation

Socializing after a urinary stoma is a process that generates psychological distress due to the difficulty of handling the device. There is also physical distress from urinary incontinence after treatment, which leads to loss of self-esteem and increased social isolation^(18)^. Men with ostomies face challenges managing their pouch in public, which limits their leisure activities, especially outside the home. Dependence on other people for transportation, difficulty finding restrooms, and discomfort with the pouch make simple activities like going out to a restaurant or attending events difficult. These physical and emotional barriers make it difficult to maintain an active and spontaneous social life^(20)^.

### Social limitations and strategies for living with a stoma in society

The change in body image has led men to keep their bodies covered, using strategies to camouflage the appearance of the collection bag and stoma, such as wearing larger clothing. This behavior reflects an attempt to preserve privacy and avoid prying eyes or judgment^(9)^. The stoma has limited travel and leisure activities such as swimming, running, and gardening. To cope, some men have adopted individual strategies, such as carrying extra clothes when out and about, staying close to bathrooms^(19)^, fasting to prevent the stoma from becoming productive^(9)^, and adopting different approaches to social interaction, from sharing every detail about the stoma to being more reserved^(19,22)^.

### Challenges in sexual performance

From sexuality’s perspective, male sociability causes physical pain during sexual activity, the perception of sexual drive as something inherent to masculinity (an obligation), and is impacted by erectile dysfunction, discomfort associated with odors and gases, fear and concern about odor and leakage, as well as shyness and shame when establishing emotional bonds^(9,21,22)^.

### Alternating male behaviors within the family structure

Family sociability is also threatened, as men tend to hide their difficulties in coping with the colostomy from family members, isolating themselves during the illness process. Social support from the wife connotes childish dependence due to deviations from self-sufficiency and virility^(22)^. Added to this is the pressure to support the family, which, faced with financial and bureaucratic obstacles due to the illness, intensifies feelings of inadequacy and failure. These elements highlight subjective and social dimensions that result in isolation and psychological distress^(21)^.

## DISCUSSION

An analysis of the studies shows that, over the past 20 years, research has focused on very different topics, aiming to understand the representations of the stoma within the social process. Studies have focused on sexuality, its impact on marital relationships, the role of social support, representations of the stoma in daily life, its limitations and adaptation after surgery, ways of fulfilling social roles, and how masculinity is impacted by the limitations imposed by the procedure.

Thus, the role of men with stoma was discussed based on specific perspectives, although sociability is a complex phenomenon, involving social relationships and interactions that permeate all aspects of daily life. These relationships range from family ties to social interactions with strangers in the public sphere; therefore, the complexity behind these interactions is guided by impulses and instincts to reproduce hierarchies and power that form the structure of society^(10)^.

Based on the first-order concepts, it was possible to synthesize the secondand third-order concepts, presenting them in a unit of interpretative context, discussed below.

### Male sociability in transformation: experiences of men with ostomies

It became clear that the presence of a stoma in men’s lives has significant impacts on sociability. Adaptation to the stoma depends primarily on how they exercise their culture and interact socially, as the procedure redefines the socialization process by requiring men to conform to socially established masculine standards^(9,18-22)^. This social perception of how they should behave is a decisive factor in how men react to living with a stoma: accepting their new bodily condition is essential for integrating into an environment where their new lifestyle differs from the dominant culture of people without stomas^(27)^.

Researchers^(3)^ emphasize that the male body connects men to the world, and physical experiences are influenced by the social and cultural categories of each individual’s masculinity. This perception results from a sociocultural construction that leads individuals to understand that illness distances them from what is considered normal, placing them in the realm of the pathological, affecting their bodies and deviating them from their pre-illness social status. Thus, the experience of illness is influenced by social and economic factors, and its interpretation is permeated by culture^(28)^. Therefore, educational programs and the development of technologies focused on healthcare must reach this population.

It is necessary to understand that, after the construction of a stoma, men experience multiple weaknesses, such as altered body image, difficulty in looking at, touching, and managing the stoma, and emotional imbalance, which reflect on their perception of their role in society^(2,9)^. For many, feeling masculine is fraught with tensions, conflicts, and stigmas that permeate sociocultural issues of a patriarchal society^(10)^. Therefore, patriarchal male domination and the choice of a lifestyle that deviates from historically established tradition also influence the adoption of this behavior^(27)^.

In this meta-synthesis, in an attempt at self-protection, reducing social interaction is one of the main defense mechanisms used^(18,19,22)^. The reasons that trigger seclusion are due to the difficulty of handling and changing the collection equipment in public places, incontinence, exposure to excrement, odor, and changes in the body image of the socially valued body^(18-20)^. Therefore, sociability associated with illness has its origins in the so-called physical manifestation of the disease, referring to the bodily symptoms that individuals identify and which, when socially recognized, confirm the changes in the body, affecting their identity^(29,30)^. This interaction occurs both psychologically, with the effects that an individual exerts on others, and in the effects suffered by themselves. In other words, sociability is a simultaneous event, a living experience in which individuals interact to form a social unit^(10)^.

Consequently, these men, who have been socialized to conform to masculine standards, need to (re)learn their social interactions and redefine their role in gender relations. When they become ill, men experience new experiences of passivity, affectivity, dependence, and fragility, and the power they once exercised over themselves and others becomes a morally limiting factor, preventing them from feeling free to express their masculinity^(31)^. This occurs because the stoma directly impacts social relationships, which tend to deteriorate during the process of accepting the new body image of the man with a stoma^(9,18-22)^.

The reciprocal action between individuals also makes illness a collective phenomenon. Within the social support network, the family is highlighted in literature^(18-22)^, which indicates the need to support the creation and strengthening of socio-affective networks among male population groups in society, given the social constructions of masculinities^(32-35)^. Men tend to hide their difficulties in coping with colostomy from their families, which disrupts family and marital relationships^(20,22)^. In such a scenario, defending behaviors such as self-sufficiency makes the process of accepting and living together difficult, because counting on the wife’s help awakens a feeling of childish dependence, a situation that harms masculinity, as the man must be the protector in the marital relationship^(22)^.

Among the evidence analyzed, the ostomy interferes with the conditioning of sociability and sexual activity, due to its impact on the mental health of these men, demonstrated by anger, fear, apprehension, shyness, and shame when establishing an emotional relationship with someone^(9)^. Thus, to experience sociability after this event, men tend to develop individual strategies more appropriate to their routine. The literature^(9,19)^ highlights the practice of fasting during periods when away from home to avoid digestion of food and excretion of feces^(19)^, carrying extra clothes when away from home, and orienting oneself regarding the position of public restrooms in case of emergency^(20)^. Regarding sexual activity, men tend to prioritize sexual positions that do not involve contact with the collection bag and, in some cases, avoid sexual activity due to feelings of shame^(9)^.

Therefore, a person’s needs lead him to adopt different forms of sociability, considering the situation he finds himself in. This multifaceted interaction coexists with the concern of reflecting on his social circle the effects of living with a stoma^(18,22)^. Thus, man experiences suffering in the physical body, due to the abnormality of his physiology, and suffering in the social context, due to the limitation of sociability^(9,18-22)^. Therefore, the dynamics of sociability in the context of illness are seen as a social coping resource, both for the ill individuals and for the social agencies that influence their lives. These individuals often seek to manage the process of coping with the illness, striving to achieve self-mastery and control over it. In other words, living with a stoma requires changes in various aspects of daily life, which generate embarrassing feelings and conflicting relationships. The attempt to return to normality is the symbolic concept of honor that governs men’s expectations and actions at home and in social life^(21)^.

To further deepen future discussions on the sociability of men with stoma, it is necessary to invest in new research that explores the contexts and dynamics of social relationships in this setting, without specific biases. This involves analyzing feelings, interpersonal relationships, the role of care, emotional support, and, most importantly, the development of new social identities after stoma creation. It also involves investing in enhancing the specificities of masculinities in the context of clinical care in nursing and healthcare, better understanding the processes of transition from health, illness, and care.

### Study limitations

It is worth emphasizing that this meta-synthesis has some limitations. The analysis was restricted to references in English, Portuguese, and Spanish, and the search was conducted only in specific databases. Furthermore, only articles available in full were selected, which may have excluded some relevant studies that would have broadened the scope of the work. It is also important to note that, although international indexes were used, studies with Brazilian samples predominated. Therefore, the inclusion of empirical studies with men from other regions of the world, especially in the Eastern context, may contribute to broadening the understanding of the phenomenon in different cultures.

### Contributions to nursing

This study makes substantial contributions to the advancement of qualitative scientific knowledge on this issue, gathering evidence that can guide clinical and social decision-making in the fields of nursing and health. It can serve as a basis for teaching in nursing training, as well as in Primary Health Care and rehabilitation. Furthermore, it contributes to advances in the implementation of targeted public policies, such as those aimed at people with intestinal stoma, people with disabilities, and comprehensive men’s health.

## FINAL CONSIDERATIONS

Being a man encompasses an identity that is also socially constructed, in which masculine culture establishes a specific definition of full masculinity in a given society. Sociability is related to collective coexistence, whose interests and material content are guided by impulses and singular purposes. In this context, social interaction is seen as a mutual relationship, in which the individual exerts influence over others and is also influenced by them.

In male sociability, the symbolic concept of honor plays an important role, establishing men’s expectations and behaviors in their personal and social lives. This concept is often associated with virility, power, and hierarchy in gender relations. However, when a man begins living with a stoma, feelings of uncertainty, prejudice, and insecurity begin to emerge. This man faces a drastic change in his routine and must adapt to the procedure demands, resulting in a notable change and/or reversal of roles in social dynamics.

## Data Availability

The research data are available only upon request.
