# Supplementary material for: Sociability of male cancer patients with stoma: a qualitative metasynthesis
Source: Rev Bras Enferm. 2025 Dec 8;78(6):e20250021. doi: 10.1590/0034-7167-2025-0021 (PMC12707607; doi:10.1590/0034-7167-2025-0021)
Supplement: Translation

# Translation: Sociabilidade de pacientes oncológicos masculinos com estomia: metassíntese qualitativa

**DOI:** 10.1590/0034-7167-2025-0021pt

**Authors:** João Vitor Antunes Lins dos Santos, Ariana Luiza Rabelo, Vander Monteiro da Conceição, Luís Fernando Santos Corrêa da Silva, Anderson Reis de Sousa, Jeferson Santos Araújo

**Affiliations:** I Universidade Federal da Fronteira Sul. Erechim, Rio Grande do Sul, Brasil; II Universidade Federal da Bahia. Salvador, Bahia, Brasil; III Universidade Federal da Fronteira Sul. Chapecó, Santa Catarina, Brasil

**Keywords:** Saúde do Homem, Estomia, Comportamento Social, Neoplasias, Revisão Sistemática.

## Abstract

**Objetivos:**

sintetizar evidências de estudos qualitativos produzidos na literatura sobre a sociabilidade de homens com estomia devido ao diagnóstico de câncer.

**Métodos:**

metassíntese qualitativa realizada em sete bases de dados com estudos publicados no período de 2004 a 2024. A amostra incluiu seis estudos, analisados pelo *software* MaxQDA^®^, validados pelo *Critical Appraisal Skills Programme* e sintetizados com base no conceito de sociabilidade.

**Resultados:**

quatro temas descritivos foram encontrados: a) Desafios no desempenho da sexualidade; b) Impactos físicos e emocionais: autoestima e isolamento social; c) Limitações sociais e estratégias para conviver com o estoma em sociedade; d) Alternância de comportamentos masculinos na estrutura familiar. Esses temas foram compreendidos pelo tema analítico: Sociabilidade masculina em transformação: experiências de homens com estomia.

**Considerações Finais:**

a masculinidade culturalmente construída molda a sociabilidade dos homens, mas viver com um estoma gera insegurança, redefine papéis sociais e impacta significativamente a dinâmica das relações públicas e privadas.

## INTRODUÇÃO

Apesar dos avanços em seu tratamento, o câncer continua sendo um problema significativo para a saúde pública em todo o mundo. Em 2020, no âmbito global, houve um total de 19.292.789 novos casos da doença, com 9.958.133 mortes decorrentes^(1)^. Esse diagnóstico envolve repercussões físicas e sociais, devido aos efeitos adversos do tratamento, que transformam significativamente seus corpos e suas vidas^(2)^.

Estudo^(3)^ enfatiza que, no âmbito global, os processos históricos e culturais associados à identidade masculina estão ligados a ideais de virilidade, poder e hierarquia nas relações de gênero. Esses fatores moldam o comportamento dessa população, afastando-os da busca por cuidados preventivos. Além disso, existem as demandas sociais que compõem a essência da masculinidade e exigem a busca por autonomia, força, controle das emoções e sentimentos como critérios para a plena masculinidade do indivíduo. Entretanto, esses ideais dificultam o diagnóstico precoce de doenças silenciosas, mormente o câncer intestinal e do aparelho urinário, que muitas vezes requerem a confecção de um estoma de eliminação^(4)^. Logo, essa problemática, de grande relevância para a saúde pública, destaca a importância de ações essenciais para a atuação profissional no campo da enfermagem.

A literatura científica^(5,6)^ também tem ressaltado que as representações culturais e identitárias associadas ao câncer e à masculinidade são influenciadas pela idealização social da figura masculina, podendo afetar significativamente a maneira como os homens enfrentam e lidam com a doença, repercutindo na maneira como cuidam do corpo, mantêm suas crenças e se relacionam com a saúde. Além disso, estabelecem vínculos entre a masculinidade e o contexto cultural, na busca por um padrão idealizado de domínio hierárquico sobre outros homens e mulheres, com comportamentos que negam a corporificação do adoecimento, enfraquecimento e vulnerabilidade^(3)^. Nesse sentido, é essencial que as equipes de enfermagem estejam aptas para lidar com os aspectos relacionais de gênero nos processos saúde e doença, e manejo clínico das demandas e necessidades de saúde.

A confecção de um estoma de eliminação gera impactos físicos, psicológicos e sociais. Entre as limitações físicas nos homens, especialmente os cisgêneros, citam-se distúrbios sexuais, incluindo disfunção erétil, alterações no impulso sexual, e alterações na excitação, na libido e na fertilidade. Nas repercussões psicológicas, podem emergir vergonha, incômodo, medo, solidão, entre outros, que repercutem em aspectos sociais, como afastamento da família e do trabalho e isolamento social^(7)^.

Para os homens, esses desafios se tornam fonte de fragilidade em seu papel na família e na sociedade^(3)^, que afetam a reintegração social e incluem restrições impostas pelo procedimento, como o uso de banheiros públicos, problemas relacionados ao odor e extravasamento, além de limitações na prática de exercícios físicos e no lazer^(8)^. Diante dessa vulnerabilidade, o homem passa por transformações no corpo e na identidade ao longo de seu curso de vida, adotando comportamentos de alienação em relação ao próprio corpo decorrente do sentimento de diferença, tornando um desafio interagir com outras pessoas^(4,9)^.

Logo, a sociabilidade do homem com estomia assume um papel crucial como conceito analítico, pois é necessário compreender o fenômeno como um conjunto complexo de práticas e interações sociais que permeiam o campo social e da saúde; portanto, carece de atenção especializada dos profissionais de enfermagem e de saúde, o que justifica a realização deste estudo. A sociabilidade é compreendida como a estrutura de disposições individuais e coletivas dos agentes sociais, os sistemas simbólicos e as estruturas sociais nas quais ele está inserido. Dessa forma, vai além das interações superficiais entre os indivíduos, englobando relações de poder, lutas simbólicas, hierarquias sociais e identitárias^(10)^.

Pesquisadores^(11)^ destacam ainda a alarmante redução da sociabilidade em pessoas de meia-idade em países asiáticos e outros como Estados Unidos, França, Itália, Espanha e Austrália. Os efeitos dessa reclusão têm impacto direto na saúde humana: homens com baixos níveis de sociabilidade apresentam maior risco de mortalidade por doenças crônico-degenerativas, como o câncer. Diante desse fato, torna-se evidente que as relações sociais, as redes de apoio e o contexto social são fundamentais ao bem-estar físico e mental, influenciando as perspectivas, os comportamentos e até mesmo as oportunidades de acesso a cuidados e suporte em saúde^(4,9)^.

Embora ainda não estejam sintetizadas, existem evidências qualitativas que descrevem o fenômeno e podem auxiliar enfermeiros e profissionais de saúde a fornecer um cuidado abrangente ao homem com estoma, o que revela uma lacuna expressa no conhecimento científico, a qual pode ser avançada com os achados aqui sintetizados. Assim, diante do contexto apresentado, em que a convivência com uma estomia abrange diversos aspectos da subjetividade humana ao envolver percepções e significados individuais e coletivos, para o campo da enfermagem, pesquisas como esta podem inspirar novas abordagens no cuidado de pacientes do sexo masculino que enfrentam o câncer e a estomia, marcando uma fase transformadora em suas vidas devido à singularidade de suas condições.

## OBJETIVOS

Sintetizar as evidências de estudos qualitativos produzidos na literatura sobre a sociabilidade de homens com estomia devido ao diagnóstico de câncer.

## MÉTODOS

### Delineamento do estudo

A revisão sistemática qualitativa seguiu a metodologia de metassíntese em quatro etapas^(12)^: (1) busca abrangente e sistemática da literatura, com recuperação de estudos relevantes; (2) avaliação da qualidade dos estudos incluídos; (3) classificação dos achados; e (4) elaboração de metassíntese dos resultados. A metassíntese qualitativa é um processo rigoroso e metódico que visa identificar, abstrair e sintetizar dados qualitativos para responder a uma específica questão de pesquisa.

O guia *Enhancing Transparency in Reporting the Synthesis of Qualitative Research* foi utilizado para relatar os elementos essenciais que devem compor uma síntese de evidências qualitativas^(13)^. O protocolo desta metassíntese foi registrado no *International Prospective Register of Systematic Reviews*, sob Protocolo n^o^ CRD42024624100.

### Estratégia de busca

Para elaborar a pergunta investigativa e a estratégia de busca, foi utilizado o modelo SPIDER^(14)^. Para identificar os estudos elegíveis na literatura, foi realizada uma busca sistemática por dois pesquisadores (primeiro e último autor) independentes, em sete bases de dados, nesta ordem de acesso: *US National Library of Medicine* (PubMed); *Cummulative Index to Nursing and Allied Health Literature* (CINAHL); *Excerpta Médica dataBASE* (Embase); *Elsevier Science’s MegaSource* (*ScienceDirec*t); *Science Citation Indexes* (*Web of Science*); *American Psychological Association* (APA) PsycINFO; e Literatura Latino-Americana e do Caribe em Ciências da Saúde (LILACS). A estratégia de busca foi desenvolvida combinando os termos dos Descritores em Ciências da Saúde, *Medical Subject Headings* (MeSH), *Elsevier’s authoritative life science thesaurus* e APA *Thesaurus of Psychological Index Terms*, além de palavras-chave da temática. Os termos foram associados pelos operadores booleanos *OR* e *AND,* de acordo com a especificidade de cada base de dados. Para exemplificar, o Quadro 1 apresenta a estratégia de busca avançada na base de dados PubMed seguindo o modelo SPIDER, a partir da qual se adaptaram as demais estratégias de acordo com as especificidades de cada base de dados.

**Quadro 1:** Modelo SPIDER da operacionalização da metassíntese

Acrônimo	Exemplo	Estratégia de busca
S	Homens com estoma diagnosticados com câncer	*“Men”[MeSH] OR “male” OR “masculinity” AND “Surgical Stomas”[MeSH] OR “Ostomy”[MeSH] OR “ostomy” OR “stoma” OR “colostomy” OR “ileostomy” OR “nephrostomy” AND “Neoplasms”[MeSH] OR “cancer” OR “oncology” OR “neoplasm”*
PI	Sociabilidade	*“Social Support”[MeSH] OR “social interaction” OR “sociability” OR “social experience” OR “interpersonal relations”*
D	Métodos qualitativos	*“Focus Groups”[MeSH] OR “Focus Group” OR “Group, Focus” OR “Anthropology, Medical”[MeSH] OR “Medical Anthropology” OR “Grounded Theory”[MeSH] OR “Theory, Grounded” OR “Hermeneutics”[MeSH] OR Hermeneutic OR Ethnographic OR “ethnographic research” OR Phenomenology OR “phenomenological research” OR Narrative OR “Interviews as Topic”[MeSH] OR Interviewers OR Interviewer OR Interviewees OR “Group Interviews” OR “Group Interview” OR “Interview, Group” OR “Interviews, Group” OR “in-depth interview” OR “qualitative interview” OR “content analysis” OR “semantic analysis”*
E	Perspectiva ou experiência sobre o fenômeno	*“Experience” OR “experiences” OR “sense” OR “meaning” OR “perspectives” OR “subjectivity” OR “life experiences”*
R	Evidências qualitativas	*“Qualitative Research”[MeSH] OR “Research, Qualitative”* *OR “Qualitative studies” OR “Qualitative”* *OR “Empirical Research”[MeSH] OR “Research, Empirical”*

Assim, foi elaborada a seguinte pergunta de pesquisa: quais as evidências de estudos qualitativos sobre a experiência da sociabilidade de homens com estoma diagnosticados com câncer?

A busca foi realizada de forma independente pelo primeiro e último autor no mês de setembro de 2024, limitada ao resgate dos registros disponíveis no período de 20 anos (2004-2024) e conduzida conforme os passos propostos pelo fluxograma *Preferred Reporting Items for Systematic Reviews and Meta-Analyses* (PRISMA)^(15)^, apresentado na Figura 1.

**Figura 1:** Fluxograma da operacionalização da metassíntese segundo o Preferred Reporting Items for Systematic Reviews and Meta-Analyses

### Seleção dos estudos e critérios de inclusão e exclusão

Por meio da busca sistemática, identificaram-se 321 registros: 56 na base de dados PubMed, 33, na CINAHL, 171, na Embase, 20, na PsycINFO, cinco, na *Web of Science*, 33, na *ScienceDirect* e três, na LILACS. Duas referências foram obtidas por meio de busca assistemática. Portanto, foram capturadas 321 publicações.

Para otimizar o processo, dois pesquisadores, de forma independente (primeiro e último autor), selecionaram os estudos por meio da plataforma *Rayyan* ^®(16)^. O cegamento do processo foi assegurado, e as possíveis discordâncias foram resolvidas através de consulta a um terceiro revisor (terceiro autor). Inicialmente, os registros identificados foram carregados na plataforma e, após retirar as duplicatas, 285 foram analisados pela leitura dos títulos e resumos. Em seguida, foram aplicados os critérios de seleção, em que 164 registros foram excluídos por motivos como: estudos com métodos quantitativos, mistos (quali-quantitativas), estudos de revisão, e por não abordarem o fenômeno de interesse. Nesse processo, foram eleitos 51 registros, que foram analisados na íntegra, entre os quais foram excluídos 11 estudos por contemplarem amostra do público feminino e masculino, e 34 evidências, por não responderem à pergunta de pesquisa. Por fim, duas referências foram encontradas por outras fontes (citações dos estudos selecionados), e apenas uma foi incluída no estudo. O presente estudo incluiu referências originais escritas em português, inglês ou espanhol que abordaram a sociabilidade entre homens com estomias decorrentes de câncer no trato urinário e intestinal. Foram selecionadas publicações que utilizaram o método qualitativo e apresentaram os discursos da população masculina de forma individualizada, publicadas de janeiro de 2004 a outubro de 2024.

Foi excluída a literatura considerada cinzenta, como livros, Trabalhos de Conclusão de Curso, teses e dissertações, além de editoriais, cartas ao editor, estudos reflexivos e de revisão. Convém salientar que, ao respeitar a ordem de pesquisa das bases de dados, os registros duplos foram considerados uma única vez. Após a análise quanto à adequação aos critérios de inclusão e exclusão, seis estudos foram eleitos para a síntese final.

### Avaliação da qualidade metodológica

Seguindo os critérios do *checklist Critical Appraisal Skills Programme* ^(17)^, dois pesquisadores avaliaram independentemente a qualidade dos estudos selecionados. Após a análise, as referências foram classificadas em duas categorias: A e B. A categoria A engloba as referências com baixo risco de viés, que atenderam a nove ou mais dos dez critérios avaliados. Já a categoria B compreende as referências com risco de viés moderado que atenderam a pelo menos cinco dos dez critérios analisados. Neste processo analítico, os artigos selecionados obedeceram à maioria dos critérios estabelecidos pelo instrumento, representando um baixo viés de risco. O Quadro 2 ilustra a avaliação dos estudos selecionados por ano, organizada pelo primeiro autor. É importante enfatizar que, durante esse processo, nenhuma referência foi excluída.

**Quadro 2:** Avaliação da qualidade dos estudos incluídos segundo o Critical Appraisal Skills Programme

Questão	Araújo, 2022^(9)^	Neris, 2020^(18)^	Bigun, 2015^(19)^	Shipp, 2015^(20)^	Dázio, 2009^(21)^	Kandemi,2017^(22)^
1. Os objetivos da pesquisa estavam reportados claramente?	•	•	•	⊕	•	•
2. A metodologia qualitativa é adequada?	•	•	•	•	•	•
3. O desenho da pesquisa estava adequado para alcançar os objetivos propostos?	•	•	•	•	•	•
4. A estratégia de recrutamento foi adequada aos objetivos da pesquisa?	•	•	•	•	•	•
5. Os dados foram coletados abordando a questão de pesquisa?	•	•	•	•	•	•
6. A relação entre o pesquisador e os participantes foi devidamente considerada?	⊕	•	•	•	•	•
7. As questões éticas foram consideradas?	•	•	•	•	•	•
8. A análise dos dados foi suficientemente rigorosa?	•	•	•	⊕	•	•
9. Os resultados foram reportados claramente?	•	•	⊕	•	⊕	•
10. A pesquisa traz contribuições?	•	•	•	•	•	•
- Classificação -	A	A	A	A	A	A

*• - Sim; o - Não;* ⊕ *- Parcialmente.*

### Extração dos dados e elaboração da síntese

Para coletar os dados das evidências, empregou-se um instrumento adaptado do JBI, contemplando variáveis como título, autores, país, ano, local de publicação, fenômeno de interesse, objetivos do estudo, população e métodos^(23)^. Com base nessas informações, analisaram-se as características das evidências, em uma perspectiva abrangente, utilizando o *software MaxQDA^®^* ^(24)^. O uso desse *software* possibilitou ampliar o conhecimento, ao explorar com maior profundidade os conteúdos extraídos e construir categorias de análise inicial. As características mencionadas permitiram resumir os principais achados das pesquisas, levando em consideração suas particularidades e identificando os conceitos de primeira ordem. Em seguida, esses conceitos foram comparados e agrupados por meio de uma abordagem interpretativa, resultando nos conceitos de segunda ordem^(25)^.

Posteriormente, os resultados foram analisados com base na análise temática indutiva^(26)^, seguindo as seguintes etapas propostas: familiarização com os dados; geração de códigos; busca por temas; revisão contínua dos temas; definição dos temas; e produção de uma interpretação explicativa. Para construir a síntese interpretativa, apresentada neste estudo por meio de temas descritivos para melhor compreender as perspectivas e experiências dos homens estomizados em seu processo de sociabilidade, foram analisados os conceitos de segunda ordem e elaborada a síntese interpretativa de terceira ordem sobre o fenômeno estudado.

## RESULTADOS

A construção da presente metassíntese engloba a descrição dos resultados de seis estudos qualitativos. A amostra final foi organizada e classificada por título, país e ano de publicação, principais objetivos, população e contexto das pesquisas, como ilustra o Quadro 3.

**Quadro 3:** Caracterização dos estudos selecionados

Título	País/ano	Objetivo	Amostra	Método	Principais resultados	Avaliação de qualidade do *Critical Appraisal Skills Programme*
***Sexuality of men experiencing intestinal ostomies: stories*** ***about feelings and meanings* ^(9)^**	Brasil 2022	Apreender os sentimentos e significados da sexualidade de homens adultos com estomias intestinais.	30 homens	Estudo qualitativo, do tipo histórico-analítico e compreensivo-exploratório, analisado pelo método de história oral temática.	Homens adultos com estomias intestinais vivenciam múltiplos sentimentos vinculados à sua sexualidade, como sentimentos impulsivos, emocionais, afetivos e orientativos	A
***“What I was and what I am”: A qualitative study of survivors’ experience of urological cancer* ^(18)^**	Brasil 2020	Analisar a experiência de sobrevivência do câncer urológico sob a óptica dos homens.	Dez homens	Estudo qualitativo, com a metodologia narrativa, analisado com base nos conceitos de cultura derivados da antropologia médica interpretativa.	As narrativas revelam transições de identidade e da condição de homens saudáveis à de homens marcados pela doença. Na tentativa de preservar a masculinidade, buscavam se mostrar como homens normais, embora a sobrevivência tenha representado uma experiência de liminaridade e ruptura biográfica.	A
***A qualitative study exploring male cancer patients’ experiences with percutaneous nephrostomy* ^(19)^**	Dinamarca 2015	Descrever como a nefrostomia é percebida pelos pacientes e suas repercussões em sua vida cotidiana.	Dez homens	Estudo qualitativo, com entrevistas semi-estruturadas interpretadas com base na Teoria Fundamentada nos Dados.	O tratamento com nefrostomia reduziu a atividade física e restringiu a vida social. Os pacientes relataram limitações para realizar suas atividades sociais habituais, mencionando o vazamento e o odor como fatores limitantes.	A
***The impact of colorectal cancer on leisure*** ***participation: A narrative study* ^(20)^**	Austrália 2015	Explorar as mudanças na participação em atividades de lazer como resultado de um diagnóstico de câncer e dos tratamentos subsequentes, além dos fatores que facilitaram ou restringiram essa participação e o significado atribuído pelos participantes aos padrões de participação em lazer.	Quatro homens	Estudo qualitativo, com método narrativo analisado por meio da análise temática.	Durante o tratamento, homens priorizaram ocupações passivas em detrimento das mais ativas ou comunitárias, devido às limitações impostas pela bolsa de colostomia.	A
***The meaning of being a man with intestinal stoma due to colorectal*** ***Cancer: an anthropological approach to masculinities* ^(21)^**	Brasil 2009	Analisar os sentidos que homens com estoma intestinal atribuem à experiência da doença e do tratamento do câncer colorretal.	16 homens	Estudo qualitativo, com dados coletados por meio de observação participante e entrevistas semiestruturadas, analisados por meio de abordagem indutiva.	Os exames diagnósticos deixaram marcas corporais e emocionais, e arranhaduras em suas masculinidades. A tentativa de retomar a normalidade foi a concepção simbólica de honra que rege as expectativas e atuações dos homens no lar e na vida social.	A
***‘Sexual Problems of Patients with Urostomy:*** ***A Qualitative Study* ^(22)^**	Turquia 2017	Identificar experiências, percepções e problemas de pacientes que realizaram urostomia devido ao câncer de bexiga em relação aos efeitos da urostomia na vida sexual deles e de seus cônjuges/parceiros(as).	Dez homens	Estudo qualitativo de abordagem fenomenológica. Coletados por meio de entrevistas semiestruturadas, os dados foram codificados e organizados em temas com base na análise de conteúdo.	Homens relataram incômodo com a imagem corporal alterada pelo estoma, destacando volume sob as roupas, dificuldade para usar calças e uso constante de agasalhos. Apontaram também disfunção sexual e ausência de apoio profissional para lidar com essas questões.	A

A organização dos conceitos de primeira ordem é descrita a partir da síntese dos principais conceitos e ideias encontrados nos artigos originais. A síntese temática permitiu identificar quatro temas descritivos: (a) Desafios no desempenho da sexualidade; (b) Impactos físicos e emocionais: autoestima e isolamento social; (c) Limitações sociais e estratégias para conviver com o estoma em sociedade; (d) Alternância de comportamentos masculinos na estrutura familiar. Esses temas descritivos foram compreendidos por meio de um tema analítico intitulado “Sociabilidade masculina em transformação: experiências de homens com estomia”. A Figura 2 ilustra o processo de análise e produção dos temas.

**Figura 2:** Representação do processo de análise e produção dos temas

O tema analítico revela que as mudanças na sociabilidade masculina são vivenciadas com aflição emocional relacionada ao tratamento, reabilitação e adaptação a um novo estilo de vida. Os homens descrevem a experiência como uma perda de autonomia e virilidade, sentindo-se limitados em sua identidade social e emocional, como se estivessem perdendo os atributos tradicionalmente associados à masculinidade.

### Impactos físicos e emocionais: autoestima e isolamento social

Desempenhar a sociabilidade após o estoma urinário é um processo que gera sofrimento psíquico pela dificuldade de manuseio do dispositivo. Ocorre também o sofrimento físico da incontinência urinária após o tratamento, que acarreta a perda da autoestima e aumento do isolamento social^(18)^. Os homens com estomia enfrentam desafios na gestão da bolsa em público, o que limita suas atividades de lazer, especialmente fora de casa. A dependência de outras pessoas para transporte, a dificuldade de encontrar banheiros e o desconforto com a bolsa transformam em tarefas complicadas as atividades simples, como sair para um restaurante ou participar de eventos. Essas barreiras físicas e emocionais dificultam a manutenção de uma vida social ativa e espontânea^(20)^.

### Limitações sociais e estratégias para conviver com o estoma em sociedade

A mudança da imagem corporal fez os homens manterem seu corpo encoberto, utilizando estratégias para camuflar a aparência da bolsa coletora e do estoma, como o uso de roupas de tamanhos maiores. Esse comportamento reflete a tentativa de preservar a privacidade e evitar olhares curiosos ou julgamentos^(9)^. A estomia limitou viagens e atividades de lazer, como natação, corrida e serviços de jardinagem. Para lidar com isso, alguns homens adotaram estratégias individuais, como carregar roupas extras quando estão fora de casa, manter-se próximo a banheiros^(19)^, manter-se em jejum para evitar que o estoma fique produtivo^(9)^ e adotar diferentes abordagens na interação social, desde compartilhar todos os detalhes sobre o estoma até ser mais reservado^(19,22)^.

### Desafios no desempenho da sexualidade

No prisma da sexualidade, a sociabilidade masculina provoca dor física durante a atividade sexual, a percepção do impulso sexual como algo inerente à masculinidade (uma obrigação), e é impactada por disfunção erétil, desconforto associado a odores e gases, medo e preocupação com odor e vazamento, além de timidez e vergonha ao estabelecer vínculos afetivos^(9,21,22)^.

### Alternância de comportamentos masculinos na estrutura familiar

A sociabilidade com a família também é ameaçada, pois os homens tendem a esconder dos familiares a dificuldade em lidar com a colostomia, isolando-se no processo de adoecimento. O suporte social na figura da esposa tem conotação de dependência infantil devido aos desvios da autossuficiência e da virilidade^(22)^. Soma-se a isso a pressão para sustentar a família que, diante de entraves financeiros e burocráticos pelo adoecimento, intensifica o sentimento de inadequação e fracasso. Esses elementos evidenciam dimensões subjetivas e sociais que resultam em isolamento e sofrimento psíquico^(21)^.

## DISCUSSÃO

A análise dos estudos evidencia que, ao longo dos últimos 20 anos, as pesquisas focaram seus objetivos em temas bastante distintos, com o intuito de compreender as representações do estoma diante do processo de sociabilidade. Os estudos se voltaram para a sexualidade, impactos na relação conjugal, figura do apoio social, representações da estomia na vida cotidiana, suas limitações e forma de adaptação após a intervenção cirúrgica, modo de desempenhar o papel social, e como a masculinidade é impactada diante das limitações impostas pelo procedimento.

Assim, o papel do homem com estoma foi discutido com base em recortes específicos, embora a sociabilidade seja um fenômeno complexo, envolvendo relações e interações sociais que permeiam todos os aspectos da vida cotidiana. Essas relações se estendem desde os laços familiares até as interações sociais com pessoas desconhecidas na esfera pública; portanto, a complexidade por trás das interações é guiada por impulsos e instintos para reproduzir hierarquias e poder que formam a estrutura da sociedade^(10)^.

Com base nos conceitos de primeira ordem, foi possível sintetizar os conceitos de segunda e terceira ordem, apresentaando-os em uma unidade de contexto interpretativo, discutido a seguir.

### Sociabilidade masculina em transformação: experiências de homens com estomia

Ficou evidente que a presença de uma estomia na vida dos homens gera impactos significativos na sociabilidade. A adaptação ao estoma depende mormente da forma como eles exercem sua cultura e interagem socialmente, porquanto o procedimento redefine o processo de socialização ao exigir que os homens se ajustem aos padrões masculinos estabelecidos socialmente^(9,18-22)^. Essa percepção social de como deve comportar-se é fator decisivo na forma como os homens reagem à convivência com o estoma: aceitar sua nova condição corporal é essencial para se integrar em um ambiente onde seu novo estilo de vida difere da cultura dominante de pessoas sem estomas^(27)^.

Pesquisadores^(3)^ enfatizam que o corpo masculino conecta o homem ao mundo, e as experiências físicas são influenciadas pelas categorias sociais e culturais da masculinidade própria de cada indivíduo. Essa percepção resulta de uma construção sociocultural que leva o indivíduo a compreender que o adoecimento o afasta do que é considerado normal, colocando-o na esfera do patológico, afetando seu corpo e o desviando de seu *status* social de poder anterior ao adoecimento. Dessa forma, a experiência de adoecer é influenciada por fatores sociais e econômicos, e sua interpretação é permeada pela cultura^(28)^. Logo, programas educativos e o desenvolvimento de tecnologias focadas no cuidado em saúde devem alcançar essa população.

É necessário compreender que, após a construção de um estoma, o homem experiencia múltiplas fragilidades, como imagem corporal alterada, dificuldade em olhar, tocar e manejar o estoma, e desequilíbrio emocional, que refletem na percepção do seu papel na sociedade^(2,9)^, porquanto, para muitos, sentir-se masculino é eivado de tensões, conflitos e estigmas que permeiam questões socioculturais de uma sociedade patriarcal^(10)^. Portanto, a dominação masculina patriarcal e a escolha de um estilo de vida que se desvia da tradição historicamente estabelecida também influem na adoção desse comportamento^(27)^.

Nesta metassíntese, na tentativa de autoproteção, a redução da interação social se apresenta como um dos principais mecanismos de defesa utilizados^(18,19,22)^. Os motivos desencadeadores da reclusão se devem à dificuldade de manuseio e troca do equipamento coletor em locais públicos, à incontinência, à exposição dos excrementos, ao odor e à alteração da imagem corporal do corpo valorizado socialmente^(18-20)^. Portanto, a sociabilidade associada à doença tem sua origem na denominada manifestação física da doença, referindo-se aos sintomas corporais que os indivíduos identificam e que, quando reconhecidos socialmente, confirmam as alterações no corpo, afetando a sua identidade^(29,30)^. Essa interação ocorre tanto no aspecto psicológico, com os efeitos que um indivíduo exerce sobre os outros, quanto nos efeitos sofridos por si próprio. Ou seja, a sociabilidade é um acontecimento simultâneo, uma experiência viva na qual os indivíduos interagem para formar uma unidade social^(10)^.

Consequentemente, esses homens, que foram socializados para se adequarem aos padrões masculinos, precisam (re)aprender suas interações sociais e redefinir seu papel nas relações de gênero. Quando adoecem, os homens vivenciam novas experiências de passividade, afetividade, dependência e fragilidade, e o poder que exerciam sobre si e sobre os outros se torna um fator moralmente limitante, o que impede que se sintam livres para expressar sua masculinidade^(31)^. Isso ocorre porque o estoma exerce um impacto direto nas relações sociais, que tendem a se deteriorar durante o processo de aceitação da nova imagem corporal do homem com estomia^(9,18-22)^.

A ação recíproca entre os indivíduos torna o adoecer também um fenômeno coletivo. Na teia do suporte social, a família é destacada na literatura^(18-22)^, o que indica a necessidade do apoio à criação e ao fortalecimento das redes socioafetivas entre os grupos populacionais masculinos na sociedade em face das construções sociais das masculinidades^(32-35)^. Os homens tendem a esconder de seus familiares a dificuldade em lidar com a colostomia, o que gera um abalo na relação familiar e conjugal^(20,22)^. Em tal cenário, a defesa de comportamentos como a autossuficiência dificulta o processo de aceitar e conviver, porque contar com o auxílio da esposa desperta uma sensação de dependência infantil, uma situação que fere a masculinidade, pois o homem deve ser o protetor na relação conjugal^(22)^.

Entre as evidências analisadas, a estomia interfere no condicionamento da sociabilidade e da atividade sexual, por sua repercussão no estado de saúde mental desse homem, demonstrado por ira, medo, receio, timidez e vergonha ao estabelecer relação afetiva com alguém^(9)^. Assim, para vivenciar a sociabilidade após esse evento, o homem tende a desenvolver estratégias individuais mais adequadas à sua rotina. A literatura^(9,19)^ destaca a prática de jejuar durante os períodos em que estão fora de casa para evitar a digestão dos alimentos e excreção das fezes^(19)^, levar roupas extras quando estiver fora de casa, e situar-se quanto à posição de banheiros públicos em caso de emergência^(20)^. Quanto à atividade sexual, o homem tende a priorizar posições sexuais que não envolvem contato com a bolsa coletora e, em alguns casos, evitar a atividade sexual, devido ao sentimento de vergonha^(9)^.

Portanto, as necessidades do homem o fazem assumir diferentes formas de sociabilidade, considerando a situação em que se encontra. Essa interação multifacetada coexiste com a preocupação de refletir em seu círculo social os efeitos de conviver com um estoma^(18,22)^. Assim, o homem experimenta sofrimento no corpo físico, pela anormalidade de sua fisiologia, e sofrimento no contexto social, pela limitação da sociabilidade^(9,18-22)^. Por isso, a dinâmica da sociabilidade no contexto do adoecimento é vista como um recurso social de enfrentamento, tanto para os indivíduos doentes quanto para as agências sociais que exercem influência em sua vida. Com frequência, esse indivíduo busca gerenciar o processo de lidar com a doença, procurando alcançar o autodomínio e o controle sobre ela. Ou seja, viver com o estoma requer mudanças em vários aspectos do cotidiano, que geram sentimentos constrangedores e relações conflituosas, e a tentativa de retomar a normalidade é a concepção simbólica de honra que rege as expectativas e atuações do homem no lar e na vida social^(21)^.

Para aprofundar as discussões futuras sobre a sociabilidade do homem com estoma, é necessário investir em novas pesquisas que explorem os contextos e as dinâmicas das relações sociais nesse cenário, sem recortes específicos. Isso envolve analisar os sentimentos, as relações interpessoais, o papel do cuidado, o suporte emocional e, principalmente, o desenvolvimento de novas identidades sociais após a confecção do estoma. Também envolve investir no incremento das especificidades das masculinidades no contexto do cuidado clínico em enfermagem e saúde, compreendendo melhor os processos de transição de saúde, doença e cuidado.

### Limitações do estudo

É oportuno enfatizar que esta metassíntese apresenta algumas limitações. A análise foi restrita a referências em inglês, português e espanhol, e a busca foi realizada apenas em bases de dados específicas. Além disso, foram selecionados somente artigos disponíveis na íntegra, o que pode ter excluído alguns estudos relevantes que ampliariam a abrangência do trabalho. Também é importante salientar que, embora tenha utilizado indexadores internacionais, predominaram estudos com amostras brasileiras. Portanto, a inclusão de estudos empíricos com homens de outras regiões do mundo, especialmente no contexto oriental, pode contribuir para ampliar a compreensão do fenômeno em diferentes culturas.

### Contribuições para a área da enfermagem

O presente estudo aporta contribuições substanciais para o avanço no conhecimento científico de escopo qualitativo acerca da problemática, reunindo evidências que podem guiar a tomada de decisão clínica e social no campo da enfermagem e saúde. Pode servir como base para o ensino na formação de enfermeiros, assim como na atuação na Atenção Primária à Saúde e na reabilitação. Além disso, contribui para avanços na implantação de políticas públicas focais, como aquelas direcionadas à pessoa com estoma intestinal, pessoa com deficiência e saúde integral masculina.

## CONSIDERAÇÕES FINAIS

Ser homem abrange uma identidade que também é construída socialmente, na qual a cultura masculina estabelece uma definição específica de masculinidade plena em dada sociedade. A sociabilidade está relacionada à convivência coletiva, cujos interesses e conteúdos materiais são guiados por impulsos e com finalidades singulares. Nesse contexto, a interação social é vista como uma relação mútua, na qual o indivíduo exerce influência sobre os outros e também é influenciado por eles.

No caso da sociabilidade masculina, a concepção simbólica de honra desempenha importante papel, estabelecendo as expectativas e comportamentos do homem em sua vida pessoal e social. Essa concepção muitas vezes está associada à virilidade, ao poder e à hierarquia nas relações de gênero, mas quando passa a conviver com um estoma, iniciam-se os sentimentos de incerteza, preconceito e insegurança, pois esse homem enfrenta uma mudança drástica em sua rotina e precisa se adaptar às imposições do procedimento, resultando em notável alteração e/ou inversão de papéis na dinâmica social.

## Data Availability

Os dados de pesquisa só estão disponíveis mediante solicitação.
